# Differences between health technology assessment topics in high- and middle-income countries: a scoping review

**DOI:** 10.1186/s13690-021-00754-6

**Published:** 2021-12-14

**Authors:** Sepehr Ghazinoory, Basireh Majidi, Shohreh Nasri, Mohammad Ehsan Zandi, Hosein Farrokhi, Majid Javedani, Majid Barzanouni

**Affiliations:** 1 Department of Information Technology Management, Tarbiat Modares University, Tehran, Iran; 2 University of Tehran, Tehran, Iran; 3 National Research Institution for Science Policy, Tehran, Iran

**Keywords:** Health technology assessment, HTA, Scoping review, High- and middle-income countries

## Abstract

**Background:**

The Health Technology Assessment (HTA) has encountered different issues and challenges over the last two decades. The main purpose of this research is to review the issues and challenges in high- and middle-income countries through reviewing studies related to the HTA.

**Methods:**

The HTA area literature of different countries was collected from 2009 to 2020 and analyzed using scoping review, based on Scopus and WoS databases.

**Results:**

Given the fact that the HTA is practically done in high- and middle-income countries, the results of reviewing the studies and articles of countries reveal that high-income countries seek to increase the participation of stakeholders and enhance the transparency of processes, policy-making, and regulation of the HTA, as well as the systematization of various participant institutions in this area. Middle-income countries, on the other hand, are mostly involved in raising awareness, training and skill development of HTA-related staff, institutionalizing the concept of HTA, and allocating appropriate resources for effective and safe decision-making in their health system.

**Conclusion:**

The problem of incoordination between stakeholders (participant institutions) in the HTA, and thereby, problems in decision-making were found in many of the studied reports and articles. Thus, one of the useful efforts to be made by different countries to maintain the integrity of this system would be the process of involving all members of this system and the formation of a healthy ecosystem in the HTA.

## Background

The significance of the health sector has been highlighted more than ever in recent years due to the increased threats of infectious and non-communicable diseases. In particular, with the onset of the COVID-19 pandemic in 2020, the area of health has turned into a priority for the world. The importance of this issue can be realized by considering the cost overrun of the healthcare and treatment area, as well as the per capita cost in this area. Data released on the costs of healthcare and treatment in various countries around the world indicate the rise of these costs more than the economic growth of countries. On the other hand, the increasing advancement of technologies in the world has led to the emergence of several technological solutions to solve healthcare-related problems. The industry and its major communication and information technologies have nowadays totally changed both the service sector and the world of production. This especially applies to the health sector where the Internet of things, cloud computing, and big data technologies have revolutionized the healthcare system and its entire ecosystem, leading it toward health care measures [1]. Technological solutions appear as an important stimulus in reducing the challenges of epidemics. However, the use of these solutions requires building trust and transparency for the effective management of health care requirements [2], which will be associated with a growing trend in the significance of technologies in the healthcare sector.

The importance of health technology assessment (HTA) will apparently increase in the future due to the multiplicity of technological choices and the breadth of usable technologies in the field of health and treatment. This implies that relying on HTA results allows achieving more efficient and effective solutions by generating knowledge and optimizing the decisions of policymakers and experts. However, making decisions, especially regarding the choice of expensive technologies, needs to be evidence-based given the scarcity of resources in the health sector [3]. This has led many countries to consider a mechanism for the rational introduction and the use of these technologies to, on one hand, control the costs, prevent their excessive rise, and optimally allocate these budgets, and on the other hand, to prevent the involvement of technologies with low safety and low effectiveness. HTA seems to be the most common tool to achieve this goal in many developed countries.

HTA is in fact a set of techniques designed to help decision-makers to incorporate technology in influencing the health of countries [4]. According to the definition by the World Health Organization (WHO) [5], HTA a systematic approach to evaluating the characteristics, effects, and impacts of technologies or policy interventions.

Reviewing the literature in this field, it can be concluded that the field of HTA has a very broad literature, indicating that it has considerably affected the generation of knowledge, dissemination, politics, behavior, or especially health promotion of countries [6, 7].

Murphy et al., 1998 suggested that the goal of all HTA research should focus on the generation of knowledge and the creation of findings that would help policymakers and stakeholders. HTA research findings directly affect the decision-making institutions and indirectly help to improve the quality of clinical practice in the national health system [7].

HTA is used as a tool to improve evidence-based policy-making and decision-making in many studies [8]. According to a report by the WHO, a shortage of qualified human resources was the main obstacle in many countries to the implementation of the process and the use of HTA since most countries lacked an academic or training program to build the HTA implementation capacity [5].

Mathew [9] mentions that most HTA reports are limited to examining the efficiency, safety, and cost-effectiveness of technology and some of them also address moral, legal, and social issues. However, HTA reports should also ideally cover other decision-making components. The integration of the desired technology into the current health system, the stability required to provide the health technology needed for all eligible individuals, the waiting time range for the technology to work or fail, the degree of novelty of the technology and new knowledge capacity influencing the decisions, and measuring the potential impact of rejecting this technology are generally some examples in this regard.

The HTA-based decisions can indeed pave the road only if there is already some kind of universal health coverage and the decision-makers are willing and able to use the HTA process by their health care systems. This is used as a model by many European countries, and some related institutions have been developed to guide the government in making decisions regarding the provision or non-provision of national health services accordingly.

Through a review of the literature in the field of HTA, this paper seeks to find out the issues and challenges faced by HTA studies in different countries. According to the methodology described in the second section of the article, a scoping review approach was used to explore and expand the evidence and map, and summarize the results of various articles published about different countries, which were extracted and analyzed from two databases and the reports of the WHO. The analysis results are provided in the third part (Results) of this article. Finally, the discussion and conclusion of the article will be presented in the fourth section.

## Methodology

In this research, the scoping review approach is used based on the framework developed by Arksey and O’Malley, which outlines a rigorous way of transparency enabling replication of the search strategy and increasing the reliability of the study findings [10]..

A “scoping review” refers to a quick review of key concepts in a particular research topic, aiming at finding the main sources and different types of evidence concerning that topic. This review focuses on evidence and relies on summarizing and describing an existing or emerging field of research [11].

This research has conducted in 2021 and summarizes and maps HTA studies conducted in different countries from 2009 to 2020. This period of years was chosen since HTA studies conducted before 2009 were in preliminary stages in most countries without relying on data. Our aim was to find out about the issues and challenges faced by different countries in the study of HTA in this period of time. The scoping review method can help to find such evidence to answer the research question. However, these studies will enter a new paradigm with the emergence of new challenges, especially with the emergence of the COVID-19 pandemic at the beginning of 2020, and countries will encounter new and different issues in HTA. Thus, new evidence will definitely emerge in the HTA of countries in the coming years and far more new questions will arise in this area of study. Accordingly, one of the topics for future research could be that “How HTA studies will vary since 2020 with the advent of COVID-19 pandemic in comparison with previous courses?

### Search strategy

**Fig. 1:**
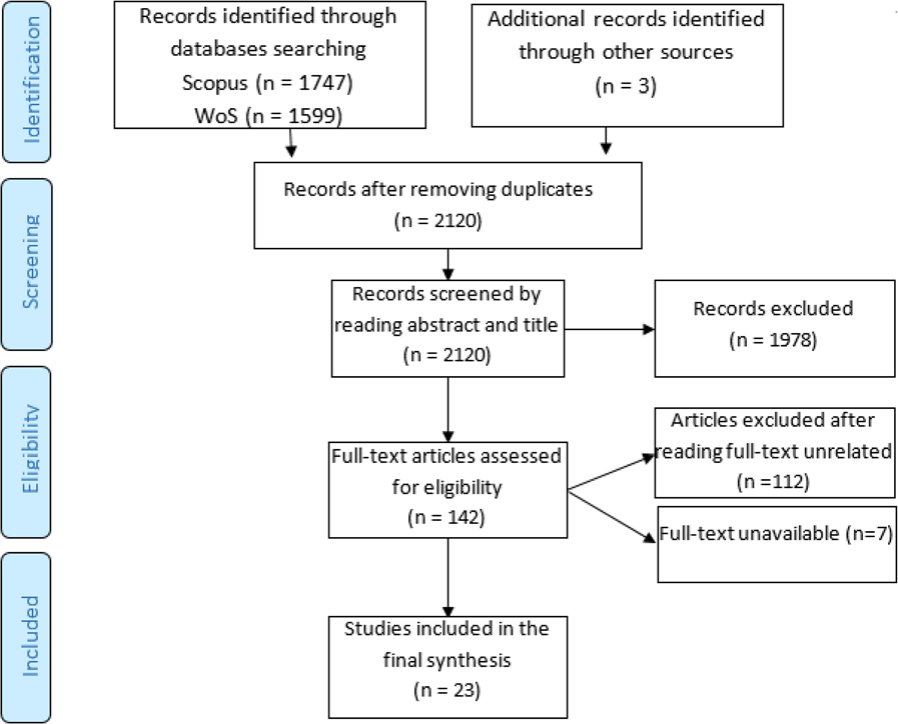
Flowchart of the literature search and screening based on the PRISMA 2009 Flow Diagram [12]

**Fig. 2:**
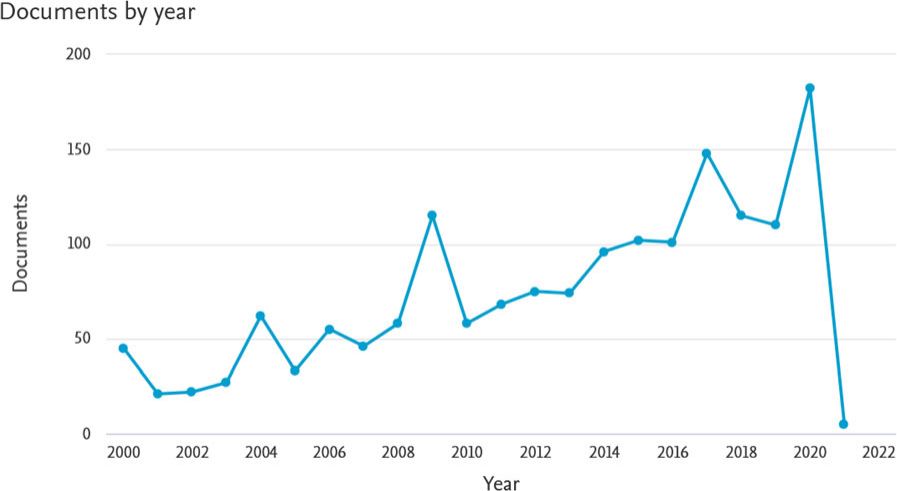
The number of articles published in the field of health technology assessment during the last two decades based on the Scopus database (Source: Authors’ elaboration)

The online Scopus and WoS databases were searched using the Boolean operators of keywords, i.e. “health” or “health assessment” or “assessment” and “technology” and “health” or “technology assessment”. In the next stage, keywords of “European”, “Asia”, “Latin America”, and “Africa” were added to the HTA and searched in each of the two databases. The main search strategy was “Health Technology Assessment”, in addition to the names of countries that published HTA studies, which were used in the search field. The search was first set to the “Title” and “Keyword” fields. The results identified a large number of articles with often little relation to the research question. Thus, the search was set to the “Abstract” field in the next step and the output provided a more limited and relevant database. Finally, the texts of the articles were excluded and only 23 articles remained to answer the research question. The research flowchart was drawn based on the PRISMA flowchart (Fig. 1). Also the number of articles published in the field of health technology assessment during the last two decades is shown in Fig. 2.

### Inclusion criteria for articles

The first step of the search retrieved 3349 articles, 1229 of which were excluded due to duplication. The titles and abstracts were the exclusion criteria of articles in the second stage. Accordingly, articles with irrelevant titles and abstracts were omitted from the review (two reviewers, review the articles and those in which both reviewers agreed to remove them were excluded). In the next stage, full texts of the articles were read to remove those that did not examine HTA in the countries. There were also articles among the reviewed studies that evaluated HTA in a very small part of a country, such as a disease in a clinic, or examined HTA in a ward of a hospital; thus, such irrelevant articles were also excluded due to incorrect generalization to the results. Since the HTA studies were generally reviewed in all parts of each country, only the texts of articles were selected that examined the general HTA in that country. Moreover, the texts of some articles were not available.

### Data extraction

The criterion for data extraction from countries was determined by designing a checklist consisting of the name of the country, level of per capita income, year of study, subject and challenge of HTA, data collection method, and obtained findings. Classification of the countries based on per capita income levels helped to identify the issues and challenges in middle- and high-income countries in terms of HTA studies. The data collection method describes the methods for obtaining HTA information in different countries. Finally, the results were extracted from those drawn by countries for HTA. In the data extraction table, a column is presented with the title of suggestions from each source for future trends in HTA. The latest report of the WHO concerning the HTA of high- and middle-income countries was also used in extracting the national data [5].

### Findings

Over the past decades, health sectors in many countries have come to the need for introducing new methods of HTA and meeting the relevant demands. This important issue is also seen in the process of articles published in this area during the last two decades.

**Fig. 3:**
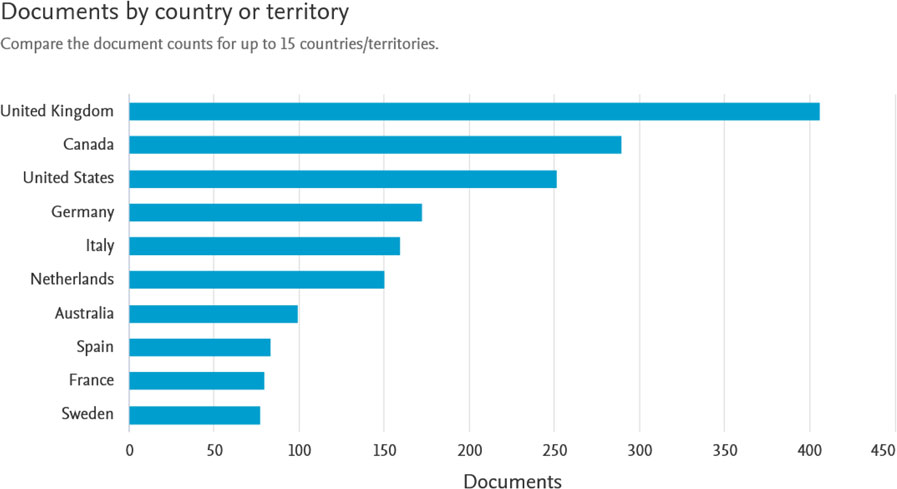
The documents published by developed countries in the field of health technology assessment based on the Scopus database (Source: Authors’ elaboration)

**Fig. 4:**
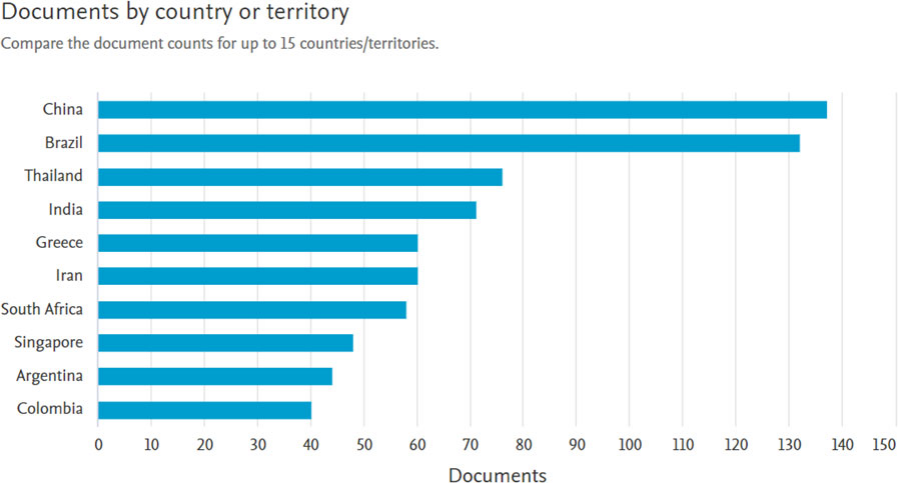
The documents published by Developing countries in the field of health technology assessment based on the Scopus database (Source: Authors’ elaboration)

HTA is primarily performed and used in high- and middle-income countries since it requires a lot of resources. Hence, HTA cannot be used and made in many countries (including the African countries) due to low income levels and lack of the required resources. As a result, these countries do not own a health technology system and no data are found from them in the reports of the WHO. Accordingly, high-income countries have the most published documents in this area (Figs. 3 and 4).

According to the WHO report in 2015 [5], countries use HTA for various goals based on their income levels. In this regard, apart from the reports by the WHO, the issue of HTA has been addressed extensively in a variety of the reviewed studies from various perspectives and aspects around the world.

According to the articles reviewed in this study and the conclusion made from the reports by the WHO regarding countries, it can be argued that the major recommendations of most researches are that the HTA should follow clearer and more explicit methods and report the results containing certain standards. For example, our review study revealed that the results reported by the reviewed articles covered a range of topics, as shown in Table 1.

**Table 1:** Various issues addressed in the HTA system review reports of the health area

Topics	References
Description of the health system	[13–15]
Review of health assessment policies	[13], [16]
Prioritization of the healthcare system sections	[17, 18]
Allocation and optimization of resources	[19, 20]
Cooperation between the agencies involved in the health assessment system	[20–23]
Procurement and supply of medicine and legislation in this area	[13, 17, 18, 24–26]
Safety, effectiveness, efficiency, and economic efficiency criteria of health sector technologies	[17, 18]
Policies and criteria for technology selection and transfer	[27–30]
Innovation	[24, 31–33]
Coverage, refunding, and pricing plans	[31]
Health data management	[21, 22, 31, 34]

On the other hand, according to the reviews made in this study and as previously reflected in the reports of WHO, the focus on improving HTA processes is through higher participation of the stakeholders, increasing the transparency of processes, and optimizing the resource allocation in most high-income countries. In middle-income countries (Latin America), however, the focus is mostly on increasing the capacity building and standardization of methods/development of guidelines.

For example, Italy has set some processes aiming at managing legislations in the pharmaceutical industry, medical services and equipment, and service location, as well as controlling the providers of personal services and coverage-related policies. In Mexico, on the other hand, studies have focused on implementing strategies to update the table and catalog of essential supplies of the healthcare sector as a supervisory tool and to ensure the establishment of adequate and necessary criteria for safety, and effectiveness. At the same time, they seek the efficiency and development of a resource coding system, standardization of technology purchasing policies, and optimization of government resources. This, in turn, reveals the difference in priorities between developed and developing countries.

On the other hand, these studies present some recommendations to overcome the challenges faced by countries in implementing the HTA system, as summarized in Table 2.

**Table 2:** The summary of research results and extracted recommendations to overcome the challenges of the health technology assessment system

Results and recommendations	References
Paying attention to legislation aimed at the development of healthcare sector technologies	[13]
Recommendation for an active approach of countries instead of passive approaches	[13]
Trying to clarify the positive effects of assessing the healthcare area technologies aimed at attracting the support of policymakers and allocating resources	[17, 18]
Investing and allocating more resources	[20]
Transparency of the budget allocation process	[21, 22]
Management of local and national information and data and utilizing data analysis in decision-making and resource allocation	[21, 22, 31]
The clarity of governments’ commitments	[24]
Continuous improvement of evaluation processes	[30, 34, 35]
Integration of assessment processes with other systems such as insurance	[27, 28, 31, 36]
Paying attention to training courses to raise the awareness of the healthcare system experts concerning the concepts, tools, and processes of the assessment system	[29]
Trying to raise the awareness of the healthcare area stakeholders and encouraging them for participation	[31]
Defining the participation process of people and the public in the health technology assessment system	[26]
The technology assessment system supporting technological innovations in the healthcare sector	[37]

The WHO categorizes the challenges of Asian countries in establishing an HTA system as follows [5]:

The first challenge is that economic data are generally not available locally. The second and perhaps the most important challenge is that most health systems in Asian countries lack enough trained personnel to perform robust economic analyses. Although this shortcoming is fixed rapidly, there are still concerns about the impact of stakeholders on understanding, interpretation, and use of economic analyses, which can result in undesirable consequences. Thus, the third challenge would be the unfamiliarity of decision-makers with accurate differences in economic analyses, which will cause a delay in the use of appropriate health technologies.

To further explain these results, an example of our analysis is provided in Table 3. The classification of countries based on middle- and high-income ones is derived from the World Bank country classifications by income level [38].^1^ According to the table, most high-income countries are engaged in issues such as policy-making and optimal decision-making in HTA, integrating the involved institutions, and increasing the knowledge and skills in this area. In contrast, middle-income countries are generally involved in topics of raising awareness and learning from the experiences of developed countries, optimal resource allocation due to lack of resources, and establishing centralized and integrated institutions in this regard. These results are summarized in Table 4.

**Table 3:** The analysis results of reports and articles that had presented the HTA in different countries

Country’s name	Country’s income level	Year of study	What part of the HTA has been addressed?	Method of collecting data from countries	Findings	Suggestions for improving HTA for the future of the country
Italy	High	2000	Processes Policies Health Technology Assessment Institutions	A descriptive statement of a member of the Italian National Research Council	Describing the health system in Italy Evaluating the control processes in health area technologies (legislations in the pharmaceutical industry, medical services and equipment, and services layout, and the control of personal providers of coverage-related services and policies Review of policies related to specific technologies (screening, remote treatment, resection, and organ and tissue transplantation) Surveying and evaluation of HTA in Italy (activities related to HTA, HTA agents, the impact of HTA)	Despite being a descriptive study, it refers to the almost uncontrolled dissemination of health technologies in different parts of Italy (without coherent policies related to health technology). Another important point mentioned in this research is the increasing sensitivity of the Italian government in the legislation of the HTA despite a passive approach to this issue.
Malaysia	High	2019	Technology assessment institutions	Summarizing the process of HTA by reviewing publicly available documents and reports as well as the experiences of the authors	HTA plays a crucial role in prioritizing treatment in public health centers in Malaysia, especially for the Ministry of Health and Medicine as the reference authority for drugs that are authorized to be prescribed in the facilities of this ministry. There are two organizations in the Ministry of Health performing the HTA as their main activities: Malaysia HTA Division and Drug Formula Management Division as a branch of Pharmacy Development and Application Division. Pharmaceutical evaluation to list the drugs is done in the Ministry of Health and Medicine under the supervision of the Formula Management Branch. The evidence-based evaluation is focused on the safety, efficacy, effectiveness, and impact of drug budgets. The cost-effectiveness is not mandatory at the moment; however, it is a concern to the decision-makers. The assessment results will be considered by the panel reviewing the list of drugs of the Ministry of Health for making decisions related to the formula.	The HTA has supported the formulary-related decisions in the Ministry of Health. The generation of evidence for being cost-effective should progress beyond efficiency or effectiveness, safety, and the budget. The impact of the HTA process is currently unknown and has not been formally evaluated up to now.
Netherlands	High	2002	Policymaking	Aimed at determining the policy relevance of the research proposals, and different methods were defined to classify, score, and weight the policy criteria by examining various classification strategies.	According to the results, different rankings of the research proposals into one of three predefined categories of high, moderate, and low relations to the policy suggest that decision-making on the budget can strongly depend on the method of choice. Also, different rankings of the research proposals using a more explicit method indicate a potential context for further development.	Considering that the budget decision-making method depends on the prioritization categories, it seems that the selection method should reflect the organizational perspective that determines the priorities.
Canada	High	2003	Challenges Policies HTA Institutions	Interviewing with key individuals related to HTA in Canada	The increasing importance and interest in HTA due to the use of economic assessments and evidence will be a significant process. The allocation of resources to HTA in Canada has been much lower than in other similar countries. The cooperation between provincial agencies and the National Coordination Office has been limited and some differences have been found regarding the role contradiction of the two in some cases. The need to allocate more resources (despite doubts about the optimality of this allocation compared to other healthcare sectors) and further efforts in the HTA process in Canada	Investing and allocating more resources to HTA, along with resolving conflicts and disputes on the use of this tool in the healthcare sector may play a role in the process of improving the health system.
Canada	High	2009	The process and challenges of developing and using HTA in the health care decisions in Canada		The decentralized nature of the healthcare system has two consequences: A close relationship with the policy-making environment, enabling to respond the requests of the HTA decision-makers according to the set time intervals. By shifting the focus of decision-making from the national to the local level, certain information needs will change to some extent.	A challenge in the process of developing and using HTA seems to be the lack of information in describing the use of HTA in Canada in the workplace, both locally and nationally. Over the years, several released reports have emphasized the lack of transparency concerning the budget decision-making process and called for greater transparency in such processes.
Data related to 19 countries	–	2019	Challenges	An extensive review of searchable documents related to the policy and publications available in English literature. The data were selected, collected, and characterized using the Arksey and O’Malley framework.	Despite discrete policies in the 19 surveyed countries, (16 African, one Central American and two Asian).^2^ most integrated community cases management programs relied largely on executors and the donors’ budget. Parallel implementation systems are often used to provide and supply drugs for integrated community cases management programs. These practices sometimes violate some of the basic principles of the healthcare system. The drug inventory depletion was still prominent in several countries and the integrated community cases management indices were sometimes not integrated into the National Health Management Information System. There were no specific incentive packages for workers with salary and wage and those without salary, associated with several regulatory challenges. Some promising measures to improve the iCCM institutionalization included community-based performance financing, use of technology with mobile devices (mHealth), small procedural improvements, and targeted and non-global service provision.	Sustainable integrated management of community affairs requires improved ownership by stakeholders and local and central governments. The government commitment should be evident in budgeting processes and executive strategies.
Tanzania	Middle	2019	Technology Assessment Institutions/Challenges	The HTA concept was examined by revising a list of national essential medicines in Tanzania by identifying a process to use HTA criteria and evidence-based decision-making in Tanzania. The use of economic evidence to make decisions was trained in the country. Then, these trainings were practically implemented to select drugs for the list of national essential drugs in Tanzania. During the revision process, capacity-building workshops were held to strengthen the HTA process.	Lessons learned from creating and strengthening processes based on transparent prioritization, especially in sub-Saharan Africa countries The HTA process was established in Tanzania from 2014 to 2018 with the establishment of the official Health Technology Assessment Committee. The committee performed successfully in the revision of the national drugs list in Tanzania. The country is institutionalizing HTA for decision-making and prioritization.	Although the use and implementation of HTA vary from country to country, some lessons have been learned that can facilitate the implementation and prioritization process based on HTA in low- and middle-income countries.
Mexico	Middle	2012	Optimizing the resources allocated to health technologies – The process of selecting health technologies – The achievements and the process of receiving new technologies	The analysis process of 394 technologies through compliance with the requirement	Implementing strategies to update the table and catalog of essential healthcare supplies (CBCISS) as a supervisory tool and ensuring sufficient and necessary criteria for safety, effectiveness, and of course, the efficiency Establishing a resource coding system, standardizing the technology purchasing policies, and optimizing the government resources	The CBCISS is some tools used by Mexico to optimize the resources allocated to the health technologies. The CBCISS goal is to help optimize the public resources through the use of technology equipment, with proven safety, therapeutic effect, and efficacy. The importance of CBCISS lies in the fact that all public institutions in the national health system should use only the technologies contained therein.
Switzerland	High	2017	The review of one of the institutions related to HTA		Analysis and evaluation of technologies in terms of suitability, cost-effectiveness, and efficiency	Developing an important program to involve all participant institutions and their cooperation to provide an optimal treatment through HTA Advising policy-makers to pay attention to the professionals abroad
United States, Canada, France, and Germany	High	2013	Policymaking		Different views were considered on the value. The patient’s health is mostly considered at the center of value. The judgment remains concerning the important decisions and is not replaced by mathematical approaches.	1. Developing a general framework to define and assess the value; developing by covered institutions/HTA and regulators 2. Disease-specific guidelines 3. Further common scientific advice to the industry regarding the proof of value 4. Developing a framework for licensing, use, and gradual repayment 5. Promoting work for a better adaptation of HTA, coverage, and procurement approaches of medical equipment
China	Middle	2009	Policies Challenges	The review of the literature and searching websites to track the growth trend of HTA in China	Initially, the transfer of HTA knowledge and the establishment of HTA units appeared to be effective methods for HTA development in China. In the late 1990s, Ministry of Health policymakers tried to integrate HTA with policy-making, aiming at improving the quality and efficiency of healthcare measures. The main government officials related to health technology include the Food and Drug Administration, the Ministry of Labor and Social Security, and the Ministry of Health, with diverse involvements in HTA. The China healthcare system has two problems: Significant waste and inefficiency as well as a serious shortage of budget (9)	An HTA-based technology license-issuing mechanism, including the license to use technology, enterprise license, and workforce license, is gradually implemented by the Ministry of Health in China. Moreover, HTA can play a crucial role in entering the technology market, insurance coverage benefits, formulation, clinical path, reimbursement, etc. A great opportunity seems for HTA to serve as a pivotal part in the healthcare reform, especially to help health sector policymakers making difficult decisions.
Finland	High	2009		Ottanen Database	The previous approach was based on intuition and results, which makes the voluntary cooperation successful and admires them with a review of the process of HTA formation in Finland since 1990, as well as the description of the network, actions, and tasks of HTA in this country.	It suggests the development of training courses for the staff of these institutions for HTA.
Japan	High	2020		Based on a review of publications and commentaries from April 2019, associated with the opinions of a group of experts on the studied major topics	It provides economic requirements for price adjustment of the list of presenting cost-effective documents to the Central Social Insurance Medical Council (Chuikyo) as a part of the Japan Ministry of Health, Labor, and Welfare for selected drugs and medical devices as well as the latest developments in HTA and the key challenges still to be resolved. Japan is the first country to adopt an algorithmic method for ICER-based pricing. There are some concerns that the implementation of HTA may reduce innovation, help increase drug delays, and ultimately negatively affects the global health system of Japan. Unlike many other countries, where HTA is designed to help with coverage or repayment decisions, HTA has been designed in Japan to help determine or adjust pricing. Based on ICER, in Japan, it can be matched to Farmacoki’s economic theory to regulate or allow the price to reflect the value. Secondly, a broader approach to value-based pricing may be considered since value measurement by merely using ICER is not enough to obtain a large drug value. Thus, the need for evidence to prove the value and support for changes in the health care system should respectively provide incentives for innovation without being evaluated commensurate with the need to produce evidence or be evaluated by suppliers or users of evidence, respectively.	Improving the HTA literacy level and ability among health professionals, universities, government, and industry should be a priority.
Asia and Oceania	Middle (Australia and New Zealand: High)	2009		Sending questionnaires to countries through the HTA Association	In the Asia-Pacific region, Australia was one of the first countries to implement official HTA programs followed by Singapore, Malaysia, and New Zealand. Other countries have also followed this program and HTA programs are currently available in China, Iran, Japan, Korea, the Philippines, Thailand, and Taiwan. HTA activities are also available in Pakistan and Indonesia, while evaluation research activities are done in India. Naturally, further efforts are required to launch HTA in countries with no such a program and to strengthen existing programs in other countries.	
International reports				Eight databases of articles and reports	Challenges that the health system should address in responsible health innovation.	For policy-makers: The justice and sustainability challenges of health systems need to be addressed preventively. Health policymakers have to turn the system-level demand “signals” into opportunities to develop innovation. Innovation policymakers need to reward the technology-based entrepreneurial activities that closely overlap with the challenges of health systems. International policy-oriented forums should share the lessons learned about innovation that better respond to the system-level challenges. For the general public: People need to know about the challenges of justice and sustainability while helping the system by articulating needs and challenges.
Asia (China, South Korea, Japan)	Middle (Japan: High)	2020	Health systems of Asian countries HTA structure and evaluation processes HTA test program and its subsequent institutionalization	Case Study of Three Country	Three case studies presented here - South Korea, Japan, and China (Table 1) – indicate the common and specific challenges of the country for the integration of HTA in existing coverage, repayment, and pricing schemes. First, developing a clear, coherent, and standardized HTA framework that explicitly defines the goals, criteria, quality standards, and the best evaluation methods and guidelines seems to be highly important. Second, the generation of country-specific real-world data should be encouraged for use in the HTAs and national databases need to be created or developed accordingly. Finally, demonstrating and measuring the useful impact of HTA on healthcare delivery, clinical outcomes, and the quality of life matters to continue the acceptance and maturity of HTA both nationally and internationally.	Challenges in China: The use of HTA is fragmented and no HTA agency is currently operating nationally to supervise and coordinate all HTA activities. HTA research is scattered throughout government, academia, and industry-based centers. The decision-making process still relies mostly on the experts’ opinions and experiences rather than on evidence-based data. HTA should be fully and formally integrated into existing pricing and refund processes. Challenges of Japan: Some of the unique features of the Japanese pricing and refund system, such as the fixed cost program, will undoubtedly make the integration of HTA into existing methods challenging for the government and manufacturers. It seems that some sort of cooperation should occur between the government and drug manufacturers. There is a need to increase internal capabilities and expertise. The key to HTA success in Japan will be the ability to produce evidence based on the national real-world data. Challenges in South Korea: The lack of specific guidelines, which initially lead to disputes between manufacturers and healthcare providers. The development of specialized staff with pharmaceutical economics expertise appears to be one of the major remaining challenges.
Latin America	Middle	2020	An overview of organizations and HTA processes in Latin America (LA) HTA schedule in Latin America	This article summarizes the discussions of participants in the 40th panel of the International Council of Pharmacy and the results of the research (ISPOR) of the panel discussion of the HTA Council for LA. An additional literature review was done to support some of the existing concepts.	This article includes a brief description of the implementation of HTA over the past 30 years and then a conceptual analysis using some examples of the broader use of HTA to support purchasing decisions and risk-sharing agreements, which may play a crucial role in determining healthcare and treatment priorities in LA in the future.	The HTA processes and methods were important in the past, although they often had separate roles (License or drug refund as examples of these cases). Having more innovative technologies and by creating value frameworks to support healthcare prioritization, HTA nowadays reveals a promising prospect for the sustainability of health systems.
Poland	High	2012	Evaluation of drugs by relevant institutions and its relationship with the interests and strategies of large multinational drug companies	A total of 109 in-depth semi-structured interviews were performed with a targeted sample of stakeholders involved in the repayment process in Poland, along with analyzing four available document sources, including the recommendations issued by AHTAPol.	Drug manufacturers used strategies on direct and indirect effects. The former included building relationships with a circle of HTA analyzers and medical professionals who were working for the agency. In indirect strategies, they employed intellectual leaders of the society in medical settings, organizations, and even political elites to support the appropriate political positions of pharmaceutical companies. An increasing proportion of drugs evaluated and recommended by AHTAPol was reported to be cost-ineffective (unaffordable) or backed by questionable pharmacological economic evidence.	Some drug evaluation results may outweigh the interests of multinational pharmaceutical companies over general payers. It was suggested that the risks involved in the drug evaluation process could be reduced through the followings: (1) The professionalization of HTA, (2) Restrictions on job search and employment after the payment of public salaries, (3) Disclosure and management of conflicts of interest of experts, (4) Patient institutionalization and public participation, and (5) Increasing the institutional separation of AHTAPol from the political elite.
Germany	High	2000	Describing the status and the development rate of Health technology regulation (HTR) according to measuring the effectiveness of decisions and their applicability	The HTA-related literature was identified by searching the literature and databases through personal contacts. The literature was analyzed according to the different sectors of the healthcare system. A national survey was performed to analyze the current state of HTA in Germany. Moreover, the names of the evaluating subjects were gathered in this study.	The results indicate that the range of HTA decisions in the outpatient ward is much more lawful and regulated than in the inpatient ward. The same is true of the dissemination and use of technology. The separation of hospitals and intensive care units seems to be an obstacle to setting the regulations and the establishment of HTA as an effective tool in Germany. Until recently, the HTA in Germany was focused on technologies such as gene technology. The German Working Group on HTA in the healthcare sector adopted a systematic approach to performing HTAs. It was concluded that the legislation of health technologies in Germany was associated with different inconsistencies, from precise legislation in the outpatient sector to almost the absence of legislation in the rehabilitation sector.	The rise in interest in HTA, along with the high priority set by the newly elected government for HTA will probably improve this situation in the future.
Europe Region Selected European countries (Group A: Germany, France, and Sweden; Group B: Poland, Bulgaria, Hungary, and Romania)	High	2018		An analytical framework between HTA methods	The evaluation criteria used in all countries include effectiveness, safety, relative efficiency, and economic data. In Group A countries, the major goals involve improving the quality of care, ensuring equal access, and efficient use of resources. Group B countries have established HTA organizations with formal guidelines, but often follow the decisions of other developed countries. They considerably emphasize the budget impact of new treatment methods and also use HTA as a tool to estimate the costs for state budgets.	HTA organizations have been developed dynamically not only in high-income countries but also in countries with limited resources. The countries being caught in the storm of its implementation can benefit from the experience and development of these countries.
Europe	High	2017	Reports of HTA institutions	A systematic approach to identifying entities involved in the HTA and selecting the evaluation reports (93 selected reports)	Clinical evidence in more than half of the studies showed moderate or low quality designed to indicate the efficacy and safety.	This study confirmed the low quality of scientific evidence used in the HTA, and thus, the use of evidence would be improved by meeting stricter quality requirements.
Switzerland	High	2017	Evaluating the effectiveness, appropriateness, economic viability, and cost-effectiveness of the HTA and recommendations to policy decision-makers and health care providers		In the area of development of processes, methods and issues related to the stakeholders’ participation, determining the processes and criteria should be raised for use in selecting the technology.	It is still in the early stages of the HTA and is supposed to continue the HTA program effectively by developing the participation of various stakeholders in this area.
India	Middle	2014	Examining and understanding the perspectives of stakeholders in the HTA in terms of evidence-based decision-making about healthcare system policy-making to evaluate their knowledge, relevant position, and interest in HTA	Semi-structured interviews with policymakers, academics, and industry experts	Although there was a good understanding of HTA among national policymakers, academics, industry experts, and the community, a lack of knowledge was seen among low-level policymakers. There was a positive perception among stakeholders for decision-making concerning the HTA generation. At the national level, however, institutions preferred to be cautious in using HTA evidence due to the very limited capacity to use evidence-based tools in the Indian health system. The key issue was the manufacturing capacity for the production and use of HTA in decision-making. Employees at the national level had little knowledge and understanding of the subject.	All stakeholders tried to promote HTA as a tool for evidence-based policy-making.

^2^Africa: Burkina Faso, Ethiopia, Ghana, Madagascar, Malawi, Mali, Mozambique, Niger, Uganda, Zambia, Cameron, Senegal, South Sudan, Rwanda, Nigeria, DRC. Central America: Nicaragua. Asia: Pakistan and Nepal

**Table 4:** The summary and conclusion on the analysis of articles and reports on the HTA in different countries

Country income	Main topics discussed in the HTA area
High-income countries	- Governance regarding regulation - Further participation of stakeholders and increasing the transparency of processes - Moving toward political learning across the borders - The emergence and continuity of national agencies performance for HTA - Following an evolutionary model and the important role of texture factors - Making decisions on the allocation of more resources - Deciding on the prioritization of technologies - Efforts for further integration and coordination of the institutions involved in the HTA - The transparency of information and data used and creation of valid and evidence-based databases - Paying attention to judgment in decision-making - Policymakers’ attention to the use of foreign knowledge and experts from other countries - Standardizing the processes of creating and using HTA - Optimal budgeting strategies - Improving the quality and reliability of the availability and efficiency of assessment methods - Increasing the stakeholders’ participation in the HTA program - The demand-orientation of the HTA system - Seeking to use efficient methods for pricing - Developing programs to improve the level of knowledge and ability of individuals and institutions involved in the HTA - Paying attention to social and ethical issues in the HTA
Middle-income countries	- Creating official HTA programs - Increasing the awareness and training of the HTA area staff - Decision-making based on the experiences of experts rather than on evidence-based data - Determining the assessment standards and guidelines - Prioritizing the HTA to reduce financial risk - Decision-making and policy-making mostly by imitating developed countries - The lack of a centralized body for regulation and oversight - The islanding of the institutions involved in the HTA - Little knowledge of the low-level policymakers - Limited resources and low capacity to use evidence-based tools in the HTA - Political challenges and incoherent policies - New establishment of the HTA and presentation of health catalogs and tables - Institutionalizing HTA in institutions and individuals involved - Serious lack of budget and significant waste of resources - The transfer of HTA-related knowledge from developed countries - The challenge of decision-making on resource allocation

Despite varying health systems around the world, this study tried to provide a list of the most important challenges ahead of countries in the establishment of an HTA system, which can serve as a guiding checklist for policymakers and stakeholders in this field. Table 4 summaries the main findings of this research about the analysis of articles and reports on the HTA in different countries.

## Conclusion

In this study, the HTA-related literature was comprehensively reviewed in the countries that published and analyzed the relevant reports. The documents published since 2009 in this area were collected and analyzed by referring to Scopus and WoS databases, as well as the reports of the WHO. The literature reviewed in this research reflected that the search strategy was designated and implemented to identify various characteristics of the HTA in different countries. Although the included articles met the inclusion criteria to evaluate the HTA in countries in the scope of our study, our research does not reflect the experiences and evidence of HTA in different health sectors.

The scoping review is used to map or configure a set of evidence; thus, our research was focused on studies indicating the “diversity” of evidence in the HTA of different countries with no in-depth concentration on the optimal choice and applicability of the HTA evidence in countries.

The role of the public and the government in the HTA of many countries seemed to be highlighted in reviewing the studies in countries, while the major role belonged to financing and budgeting. The countries in both groups encountered different challenges in HTA. In both groups, however, HTA was identified as an essential tool to improve the management of scarce resources in the health sector.

The dilemma of incoordination and segregation of participant institutions was found in the HTA process associated with resulting problems in decision-making in many of the reviewed reports and articles. It can probably be concluded that one of the useful efforts to be made by different countries to maintain the integrity of this system would be to involve all members of this system and form a health ecosystem in the HTA. As such, a large number of active actors (patients, doctors, nurses, companies and government agencies, suppliers, etc.) will develop their connections and communications with foreign actors to solve different challenges of this ecosystem.

Furthermore, the rapid growth of digital technologies (such as Big Data technologies) causes the health system of middle-income countries to face huge changes and transformations while makes the high-income countries move toward creating strategic innovation frameworks in the health ecosystem [39].

## Data Availability

Data available within the article or its supplementary materials.

## Abbreviations

HTA: Health Technology Assessment
WHO: World Health Organization

## Footnotes

1 **Table:**

^1^GNI per capita	
**Group**	July 2020
Low Income	< 1036
Lower-Middle Income	1036–4045
Upper-Middle Income	4046–12,535
High Income	> 12,535
Source: World Bank 2020	
