# Correction to: Differences between health technology assessment topics in high- and middle-income coun8tries: a scoping review

**DOI:** 10.1186/s13690-022-00808-3

**Published:** 2022-02-22

**Authors:** Sepehr Ghazinoory, Basireh Majidi, Shohreh Nasri, Mohammad Ehsan Zandi, Hosein Farrokhi, Majid Javedani, Majid Barzanouni

**Affiliations:** 1 Department of Information Technology Management, Tarbiat Modares University, Tehran, Iran; 2 University of Tehran, Tehran, Iran; 3 National Research Institution for Science Policy, Tehran, Iran

**Correction to: Arch Public Health 79, 225 (2021)**

**https://doi.org/10.1186/s13690-021-00754-6**

Following publication of the original article [1], the authors reported that the fourth author’s name needs to update from "Ehsan Zandi" to "Mohammad Ehsan Zandi".

The correct author’s name has been provided in this Correction.

The original article [1] has been updated.
